# Emergency department visits for head trauma in the United States

**DOI:** 10.1186/s12873-016-0071-8

**Published:** 2016-01-19

**Authors:** Christopher E. Gaw, Mark R. Zonfrillo

**Affiliations:** Perelman School of Medicine at the University of Pennsylvania, 3400 Civic Center Boulevard, Building 421, Philadelphia, PA 19104 USA; Department of Emergency Medicine, Alpert Medical School of Brown University, 55 Claverick St., 2nd floor, Providence, RI 02903 USA

**Keywords:** Concussion, Emergency department, Head trauma, National Electronic Injury Surveillance System, Traumatic brain injury

## Abstract

**Background:**

Head trauma affects millions of Americans each year and has significant morbidity and economic costs to society. The objective of this study is to describe the epidemiology of head traumas presenting to emergency departments in the United States.

**Methods:**

The National Electronic Injury Surveillance System-All Injury Program was queried to conduct a retrospective analysis of head traumas treated in U.S. emergency departments. 207,159 cases of nonfatal head trauma from January 1, 2007 to December 31, 2011 were included in this study.

**Results:**

An estimated 10,746,629 (95 % confidence interval: 8,368,720-13,124,537) head traumas were treated in U.S. emergency departments (EDs) during the study period, averaging 2,149,326 cases annually. The annual injury rate per 10,000 population increased from 55.2 in 2007 to 85.4 in 2011, with the largest increases seen in children ≤11 years of age and in adults >65 years of age. Traffic-related head trauma accounted for an estimated 1,819,824 visits to U.S. EDs over the study period and was associated with a 1.74 times greater risk of a hospital admission compared to injuries due to non-traffic-related causes. Assaults (95.9 %) were the most common reason for head trauma in cases where injury intent was documented, and 16.9 % of assault-related head trauma occurred in children 0-17 years of age. When analyzed separately from other head traumas, concussions increased by 37.5 % over the study period, and nearly a third (29.9 %) of all concussions were sports-related.

**Conclusions:**

The increase in the number and rate of head traumas treated in U.S. EDs warrants continued injury prevention efforts and improvements in injury nomenclature and surveillance.

## Background

Head trauma encompasses a wide variety of injuries with differing severities, ranging from trivial head wounds to traumatic brain injuries. In the United States, a large emphasis has been placed on traumatic brain injuries (TBIs); more than 1.7 million TBIs are estimated to occur each year in the U.S., 75 % of which are concussions or other mild traumatic brain injuries [1, 2]. A diverse array of injury mechanisms have been associated with TBIs, including falls, motor vehicle crashes, assaults, and sports-related injuries [2, 3]. Though many patients who suffer a mild TBI recover rapidly and fully, long-term symptoms after injury, including fatigue [4, 5], sleep disturbances [4], and memory [4, 6], have been well documented. The direct and indirect costs from TBIs to the U.S. economy have been estimated to exceed billions of dollars annually [7, 8]. Due to the associated high prevalence and costs, TBI prevention has become a public health priority in the United States in recent years [1, 9, 10].

The epidemiology of head trauma, which encompasses both TBIs as well as more minor head injuries, has not been well represented in the literature. Since patients with head trauma often utilize healthcare resources, understanding head trauma injury patterns is valuable in helping shape public policy and providing information to inform injury prevention efforts. Given that most patients who present with head trauma are often triaged and treated in an emergency department (ED) setting [1], a national emergency department dataset, such as the National Electronic Injury Surveillance System (NEISS), is a valuable data source for injury epidemiology studies. The NEISS can be used to generate estimates of injury across U.S emergency departments (EDs), allowing for the characterization of injury patterns and healthcare system burdens on a national level. However, only a few contemporary studies have utilized national emergency department datasets to examine traumatic brain injuries [11] or sports-related concussions [12].

The use of the NEISS in head trauma surveillance was identified in a report to Congress in 2003 by the Centers for Disease Control and Prevention (CDC) as a priority recommendation [1]. Its use, however, has been complicated by the lack of a standardized case definition; the NEISS does not use the *International Classification of Diseases, Ninth Revision, Clinical Modification* (ICD-9-CM) diagnosis codes in its classification of injury diagnosis [13, 14]. Recent studies have presented a working case definition for TBIs in the NEISS, which includes concussions, head fractures, and internal organ injuries to the head, that has a high sensitivity and specificity when compared to ICD-9-CM diagnosis codes [15–17]. The objective of this study is to describe the epidemiology of head trauma—focusing on secular trends and characteristics of specific modes of injury—treated in U.S. EDs among patients of all ages using the National Electronic Injury Surveillance System-All Injury Program (NEISS-AIP).

## Methods

### Study design

This study was a retrospective cohort study utilizing the National Electronic Injury Surveillance System-All Injury Program, a nationally representative database maintained by the U.S. Consumer Product Safety Commission (CPSC). This study was exempt from review by the University of Pennsylvania Institutional Review Board.

### Study setting and population

The NEISS-AIP is a collaborative database maintained jointly by the CDC and the CPSC [18]. Composed of a subset of participating NEISS hospitals, NEISS-AIP monitors all external causes of nonfatal injuries and poisonings treated in U.S. EDs. NEISS-AIP provides data on approximately over 500,000 cases annually and represents a stratified probability sample of all hospitals with a 24-hour ED with at least 6 beds in the United States and its territories [14, 18]. Professional NEISS coders abstract information from ED medical records including patient demographics, injury diagnosis, affected body region, disposition from the ED, mechanism of injury, and the intent of injury. Through weighting factors provided by the CPSC, NEISS-AIP data can be used to calculate nationally representative estimates of injuries treated nationwide. Data in this study were obtained from the Inter-university Consortium for Political and Social Research, a public data archive maintained by the University of Michigan [19].

All nonfatal head traumas reported through NEISS-AIP from January 1, 2007 to December 31, 2011 were retrospectively analyzed. Cases of head trauma were identified using the following NEISS diagnosis and body part codes in concordance with previous studies [15–17]: concussion (diagnosis code 52), fracture to the head (diagnosis code 57, body part code 75), and internal organ injury to the head (diagnosis code 62, body part code 75). In the event that multiple diagnoses are present in the ED record, the most severe injury is coded. Though previous studies label cases with these diagnosis and body part codes as TBIs, the more conservative term “head trauma” is used throughout this study, as the case definition may include non-TBI injuries in addition to TBIs [16]. A total of 207,159 cases of head trauma during the study period were analyzed. Among these cases, 38,534 cases with a concussion diagnosis (diagnosis code 52) were isolated for sub-analysis.

### Study variables

All study variables were coded by NEISS coders using a standardized data dictionary, and these variables were regrouped for further analysis in this study. The NEISS variable for age was regrouped into 6 age groups: (1) 0–11, (2) 12–17, (3) 18–24, (4) 25–44, (5) 45–64 and (6) 65+. Disposition from the ED was categorized as (1) treated and released, (2) admitted/hospitalized (including NEISS variables of treated and transferred, treated and admitted, and held for <24 hours for observation), (3) left against medical advice, or (4) not documented. The precipitating cause of injury was regrouped into (1) motor vehicle—occupant, (2) motorcyclist, (3) pedal cyclist, (4) pedestrian, (5) other transport, (6) fall, (7) struck by/against, (8) other specified, or (9) unspecified/unknown.

Perpetrator relationship in assault cases were categorized as (1) multiple, (2) friend/acquaintance, (3) spouse/partner, (4) other relative, (5) stranger, (6) other specified, and (7) unspecified/unknown. The reason for assault was regrouped as (1) altercation, (2) robbery/burglary, (3) other specified, or (4) unspecified/unknown. All analyses involving mechanism of injury utilized the precipitating cause of injury variable (the cause of injury that started the chain of events that leads to an injury) rather than the direct cause of injury, as the precipitating cause is more relevant to injury prevention efforts. The 39 individual sports categories provided by NEISS-AIP were examined, and the top 7 head trauma associated organized sports and the top 12 head trauma associated individual sports were isolated based on prevalence for further analysis.

### Statistical analysis

Data were analyzed using SPSS version 21.0 (SPSS Inc., Chicago, IL) statistical software. National injury estimates were calculated using statistical weights provided by the CPSC; each sample weight represents the inverse probability of the selection of a logged case. Analyses that involved fewer than 20 unweighted NEISS-AIP cases, 1200 weighted cases, or a coefficient of variation >30.0 % were considered unstable and were excluded from the results. All data reported in this article are national estimates unless otherwise noted. U.S. Census Bureau intercensal and postcensal population estimates from 2007 to 2011 were used to calculate injury rates for the study period [20–22]. Statistical analyses included Rao-Scott *χ*^2^ analysis to assess differences between years for secular trends and calculation of relative risk (RR) with 95 % confidence intervals (CIs).

## Results

### Sample demographics

Between 2007 and 2011, an estimated 10,746,629 (95 % CI: 8,368,720-13,124,537) patients were treated for head trauma in United States EDs, averaging 2,149,326 (95 % CI: 1,673,744-2,624,907) or 70.1 (95 % CI: 54.6-85.6) injuries per 10,000 population annually. Nearly a quarter (23.3 %) of head traumas was documented in children ≤11 years of age, and males (54.6 %) were injured more often than females (Table 1). Of the 15.7 % of head traumas that resulted in a hospitalization, over a third involved individuals over the age of 65 (35.5 %).

**Table 1:** Characteristics of head trauma treated in U.S. emergency departments

Description	Cases (n)	National estimate (%)^a^	95 % confidence interval
Gender			
Male	116,291	5,865,536 (54.6)	4,532,344–7,198,727
Female	90,850	4,880,227 (45.4)	3,801,087–5,959,367
Unknown^c^	18	^b^	^b^
Age			
0–11	64,976	2,506,502 (23.3)	1,891,605–3,121,400
12–17	28,749	1,284,749 (12.0)	1,006,639–1,562,859
18–24	22,960	1,391,571 (12.9)	1,006,590–1,776,552
25–44	34,519	2,099,395 (19.5)	1,628,127–2,570,663
45–64	27,415	1,656,661 (15.4)	1,273,498–2,039,825
65+	28,453	1,803,832 (16.8)	1,359,072–2,248,592
Unknown	87	^b^	^b^
Location			
Home	59,001	3,012,637 (28.0)	2,259,680–3,765,595
School/sports/recreation place	29,063	1,283,775 (11.9)	983,083–1,584,468
Street	36,816	2,018,501 (18.8)	1,232,527–2,804,475
Other property	26,943	1,504,002 (14.0)	1,145,785–1,862,219
Farm	340	28,406 (0.3)	13,033–43,779
Unknown	54,996	2,899,307 (27.0)	1,606,967–4,191,646
Disposition			
Treated and released	169,772	8,913,111 (82.9)	6,964,493–1,0861,729
Admitted/hospitalized	34,703	1,686,349 (15.7)	1,118,291–2,254,406
AMA	2626	144,948 (1.3)	88,526–201,370
Unknown	58	^b^	^b^
Traffic-related injury			
Yes	32,997	1,819,824 (16.9)	1,068,129–2,571,519
No	5206	297,173 (2.8)	191,099–403,247
Unknown/unspecified	168,956	8,629,631 (80.3)	6,817,644–10,441,619
Sports-related injury			
Yes	36,270 1,714,388	1,714,388 (16.0)	1,260,465–2,168,310
No	147,916	7,770,932 (72.3)	6,037,623–9,504,241
Unknown/unspecified	22,973	1,261,309 (11.7)	964,750–1,557,868

^a^Percentages may not total to 100 % due to rounding

^b^Estimate is potentially unstable since unweighted cases <20, national estimate <1200 cases, or coefficient of variation >30.0 %. No CI is provided

^c^Unknown cases are included in the total estimates of ED visits in the manuscript text

### Secular trends

**Fig. 1:**
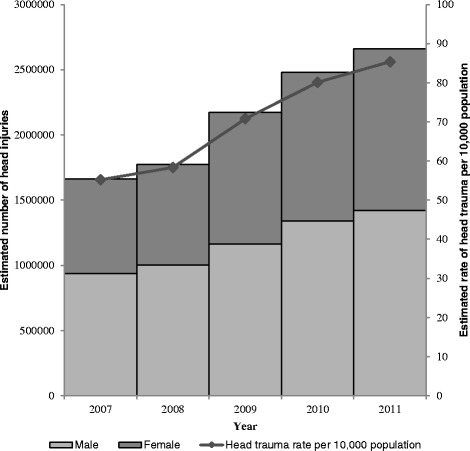
Estimated number and rate of head trauma treated in U.S. emergency departments

The annual number of head traumas diagnosed increased by 60.0 % (*χ*^2^ = 47.95, *p* < 0.001) over the study period, from 1,662,593 (95 % CI: 1,188,275-2,136,910) visits in 2007 to 2,659,942 (95 % CI: 2,040,447-3,279,437) visits in 2011 (Fig. 1). Similarly, annual injury rates per 10,000 population increased by 54.7 %, from 55.2 (95 % CI: 39.4-70.9) in 2007 to 85.4 (95 % CI: 65.5- 105.2) in 2011. The largest increases in head trauma diagnoses were documented in children ≤11 years of age and adults older than 65, with increases from 377,803 (95 % CI: 273,687-481,920) to 614,321 (95 % CI: 463,161-765,482) injuries and 244,830 (95 % CI: 181,167-307,593) to 470,660 (95 % CI: 332,008-609,311) injuries over the study period, respectively. When analyzed by gender, males experienced an increase from 937,484 (95 % CI: 650,846-1,224,122) in 2007 to 1,420,568 (95 % CI: 1,097,769-1,743,336) in 2011. Females experienced an increase from 724,932 (95 % CI: 532,714-917,150) to 1,239,293 (95 % CI: 936,391-1,542,194) over the study period.

**Fig. 2:**
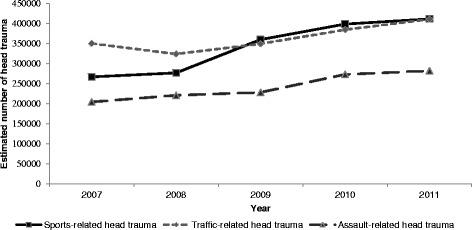
Estimated number of head trauma treated in U.S. emergency departments by injury precipitant

When divided into subcategories (Fig. 2), diagnosed head trauma increased in several areas over the study period. Sports-related head trauma experienced a 54.1 % increase, from 267,051 (95 % CI: 183,751-350,351) injuries in 2007 to 411,606 (95 % CI: 303,700-519,511) injuries in 2011. Traffic and assault-related head traumas also increased from 2007 to 2011 by 17.3 and 38.1 %, respectively.

### Traffic-related head trauma

An estimated 1,819,824 (95 % CI: 1,068,129-2,571,519) head trauma visits to U.S. EDs during the study period were documented traffic or motor vehicle crashes on highways and roadways. Nearly a third of traffic-related head traumas (31.2 %) involved 25–44 year-olds, followed by 18–24 year-olds (23.8 %). The majority of traffic-related head traumas occurred in males (55.6 %). When occupant status in the vehicle was documented, 42.8 % of traffic-related head traumas involved the driver and 23.9 % involved the passenger. An estimated 444,842 (95 % CI: 166,460-723,224) hospital admissions were associated with traffic-related head trauma, and explicitly documented traffic-related head traumas were 1.74 times (95 % CI: 1.45-2.08) more likely to lead to a hospital admission compared to other causes.

In cases of traffic-related head trauma where the precipitating cause of injury was documented, 78.0 % of all injuries occurred when the patient was in a motor vehicle (Table 2). A greater proportion of males were involved in motorcycle-related (9.4 %) and pedal-cycle-related (11.1 %) head traumas than females (2.0 and 3.4 %, respectively). Among children ≤11 years of age with a traffic-related head trauma, pedal-cycle-related and pedestrian-related traffic injuries accounted for 13.4 and 10.6 % of head traumas, respectively. In 18–24-year-olds with a traffic-related head trauma, 5.2 and 4.1 % of head traumas were attributed to pedal-cycle-related and pedestrian-related traffic injuries, respectively.

**Table 2:** Estimated number of head trauma treated in U.S. EDs incurred during traffic-related injuries with 95 % CI

Sex		Precipitating cause of injury (PCI)									
Male		MV-occupant		Motorcyclist		Pedal cyclist		Pedestrian		Other transport	
		Estimated ED visits	% of all MV-occupant (95 % CI)	Estimated ED visits	% of all motorcyclist (95 % CI)	Estimated ED visits	% of all pedal cyclist (95 % CI)	Estimated ED visits	% of all pedestrian (95 % CI)	Estimated ED visits	% of all other transport (95 % CI)
	0–11	51,387	3.6 (3.0–4.2)	^a^	^a^	13,565	9.7 (7.3–12.2)	9740	8.6 (6.2–11.0)	2476	6.8 (4.4–9.3)
	12–17	69,985	4.9 (4.3–5.6)	3,760	3.4 (2.3–4.4)	21,672	15.5 (10.0–21.1)	8943	7.9 (6.7–9.1)	3975	11.0 (8.1–13.9)
	18–24	188,740	13.3 (12.1–14.5)	20,160	18.0 (15.7–20.4)	17,415	12.5 (9.0–16.0)	10,819	9.5 (8.1–11.0)	2741	7.6 (5.0–10.2)
	25–44	233,474	16.5 (15.4–17.5)	36,201	32.4 (29.8–35.0)	26,270	18.8 (16.9–20.8)	18,420	16.2 (14.5–18.0)	4840	13.4 (10.4–16.3)
	45–64	123,525	8.7 (8.2–9.2)	29,233	26.2 (22.9–29.4)	25,797	18.5 (15.9–21.1)	15,590	13.7 (10.8–16.7)	3566	9.9 (6.7–13.1)
	65+	47,796	3.4 (3.0–3.7)	4,588	4.1 (3.0–5.2)	7818	5.6 (3.7–7.5)	4971	4.4 (3.4–5.4)	2424	6.7 (4.5–8.9)
	Total	714,907		95,052		112,537		68,483		20,022	
Female		MV-occupant		Motorcyclist		Pedal cyclist		Pedestrian		Other transport	
		Estimated ED visits	% of all MV-occupant (95 % CI)	Estimated ED visits	% of all motorcyclist (95 % CI)	Estimated ED visits	% of all pedal cyclist (95 % CI)	Estimated ED visits	% of all pedestrian (95 % CI)	Estimated ED visits	% of all other transport (95 % CI)
	0–11	44,986	3.2 (2.7–3.6)	^a^	^a^	4351	3.1 (1.7–4.6)	4482	3.9 (3.2–4.7)	1322	3.7 (1.9–5.4)
	12–17	81,698	5.8 (5.1–6.4)	^a^	^a^	3301	2.4 (1.4–3.3)	5403	4.8 (3.7–5.8)	2115	5.8 (3.5–8.2)
	18–24	176,821	12.5 (11.6–13.3)	2,599	2.3 (1.8–2.9)	5051	3.6 (1.8–5.5)	6762	6.0 (5.0–6.9)	2099	5.8 (4.3–7.3)
	25–44	218,453	15.4 (14.3–16.5)	6,639	5.9 (4.3–7.6)	7482	5.4 (3.4–7.3)	11,638	10.2 (8.2–12.3)	3686	10.2 (7.7–12.7)
	45–64	130,058	9.2 (8.3–10.0)	5,766	5.2 (4.1–6.3)	5228	3.7 (2.2–5.3)	10,509	9.3 (7.5–11.0)	3927	10.9 (8.6–13.1)
	65+	50,281	3.5 (3.1–3.9)	^a^	^a^	1636	1.2 (0.5–1.8)	5763	5.1 (3.7–6.4)	2979	8.2 (5.8–10.7)
	Total	702,297		16,396		27,049		44,557		16,128	

^a^Estimate is potentially unstable since unweighted cases <20, national estimate <1,200 cases, or coefficient of variation >30.0 %. No CI is provided

### Sports-related head trauma

1,714,388 (95 % CI: 1,260,465-2,168,310) sports-related head traumas were treated in U.S. emergency departments from 2007 to 2011. Nearly two-fifths of all sports-related head traumas (37.9 %) occurred in children 12–17 years of age, and over three-quarters (78.6 %) of all sports-related head traumas occurred in patients ≤24 years of age. Males (68.9 %) accounted for the majority of sports-related head traumas. When head traumas were stratified by the most commonly associated individual and organized sports, many have a clear male or female predominance (Table 3). Football and basketball were the two most common organized sports associated with a sports-related head trauma, accounting for 12.8 and 7.7 % of all sports-related head traumas, respectively. Bicycling and playground injuries, accounting for 16.7 and 6.8 % of all sports-related head traumas, respectively, were the most common individual sports associated with sports-related head trauma.

**Table 3:** Organized and individual sports-related head trauma by sex treated in U.S. EDs with 95 % CI

Organized sports (OS)	Male		Female	
	Estimated ED visits	% of all OS (95 % CI)	Estimated ED visits	% of all OS (95 % CI)
Football	209,946	33.3 (30.7–35.8)	10,312	1.6 (1.3–1.9)
Basketball	89,595	14.2 (12.5–15.9)	42,335	6.7 (5.8–7.7)
Soccer	54,899	8.7 (7.0–10.4)	43,811	6.9 (5.3–8.5)
Baseball	68,416	10.8 (9.6–12.0)	15,106	2.4 (1.9–2.9)
Hockey	34,109	^a^	7341	1.2 (0.6–1.7)
Softball	12,304	2.0 (1.5–2.4)	28,912	4.6 (3.9–5.2)
Volleyball	3081	0.5 (0.2–0.7)	10,767	1.7 (1.4–2.1)
Individual sports (IS)	Male		Female	
	Estimated ED visits	% of all IS (95 % CI)	Estimated ED visits	% of all IS (95 % CI)
Bicycle	221,150	25.1 (22.7–27.5)	65,828	7.5 (6.6–8.4)
Playground	63,921	7.3 (5.8–8.7)	52,023	5.9 (4.8–7.0)
ATV	56,149	6.4 (4.0–8.8)	24,542	2.8 (1.5–4.0)
Horseback	16,376	1.9 (0.9–2.8)	45,481	5.2 (3.9–6.5)
Skateboard	43,506	4.9 (3.3–6.5)	6186	0.7 (0.4–1.0)
Snow skiing	32,643	^a^	14,649	^a^
Misc ball games	31,143	3.5 (2.4–4.7)	13,963	1.6 (0.9–2.2)
Exercise	24,628	2.8 (2.3–3.3)	20,253	2.3 (1.9–2.7)
Swimming	26,392	3.0 (2.6–3.4)	18,423	2.1 (1.7–2.5)
Moped/Minibike/Other Off-road	35,314	4.0 (3.2–4.8)	4421	0.5 (0.3–0.7)
Gymnastics	5,627	0.6 (0.4–0.8)	27,638	3.1 (2.5–3.8)
Combative	26,269	3.3 (2.5–4.1)	1811	0.2 (0.1–0.3)

^a^Estimate is potentially unstable since unweighted cases <20, national estimate <1200 cases, or coefficient of variation >30.0 %. No CI is provided

Explicitly documented sports-related head traumas were associated with a 0.60 times (95 % CI: 0.51–0.71) risk of hospitalization compared to other causes. An estimated 24,978 patients (95 % CI: 15,187-34,138), or 4.0 % of all patients who participated in a top 7 organized sport, were hospitalized while 95.3 % were treated and released. In contrast, 127,837 (95 % CI: 56,893-198,783) patients (14.5 %) injured while participating in one of the 12 individual sport or recreational activities were admitted to a hospital. Bicycling (37.6 %), ATV use (22.1 %), and horseback riding (12.3 %) accounted for nearly three quarters of all individual sports-related hospital admissions.

### Head traumas associated with specific intents

Among cases where intent of injury was documented, assaults were the most common reason associated with head traumas (95.9 %), followed by legal intervention (1.1 %) (Table 4). Multiple perpetrators (38.0 %) were the most common relationship type documented in head trauma assaults, followed by an acquaintance or friend (21.6 %). Altercations (81.2 %) and robberies (12.7 %) were the most commonly cited reason for an assault-related head trauma. Nearly two-fifths of all assault-related head traumas occurred in the 25–44 year age group (38.8 %), and two-thirds of assault victims were male (66.6 %). Children less than 18 years of age accounted for 16.9 % of assault-related head traumas. Head traumas associated with legal interventions were most common among patients 25–44 years of age (48.1 %) and among male patients (89.7 %). Patients who suffered a head trauma related to a self-inflicted injury were nearly 17 times as likely (*RR* = 16.77, 95 % CI: 9.90-28.39) to be admitted to a hospital compared to other intents of injury.

**Table 4:** Characteristics of head trauma associated with specific intents treated in U.S. EDs

Description	Cases (n)	National estimate (%)^a^	95 % confidence interval
Intent			
Assault	22,010	1,209,403 (95.9)	923,354–1,495,451
Legal intervention	722	38,238 (1.1)	27,960–48,517
Self-harm	241	13,668 (3.0)	7,513–19,823
Perpetrator relationship in assault			
Multiple	4421	230,032 (38.0)	158,331–301,733
Friend/acquaintance	2520	130,650 (21.6)	100,598–160,702
Spouse/partner	2130	127,958 (21.1)	95,647–160,269
Other relative	911	48,894 (8.1)	37,968–59,819
Stranger	804	44,933 (7.4)	26,666–63,199
Other specified	531	23,771 (3.9)	17,800–29,742
Reason for assault			
Altercation	4193	225,363 (81.2)	176,632–274,095
Robbery/burglary	653	35,251 (12.7)	21,057–49,445
Other specified	394	16,928 (6.1)	7,685–26,172

^a^Percentages may not total to 100 % due to rounding

### Concussions

**Fig. 3:**
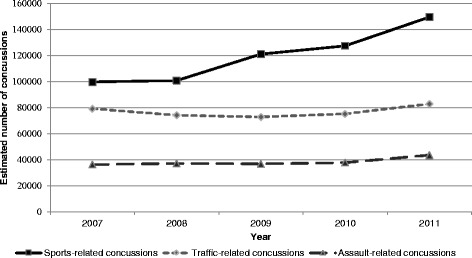
Estimated number of concussions treated in U.S. emergency departments by injury precipitant

An estimated 2,006,477 (95 % CI: 1,592,183-2,420,772) concussions occurred over the study period, averaging 401,295 (95 % CI: 318,437-484,154) concussions or 13.1 (95 % CI: 10.4-15.8) concussions per 10,000 population annually. The number of concussions increased from 344,001 (95 % CI: 249,465-438,538) in 2007 to 472,881 (95 % CI: 367,456-578,305) in 2011, an increase of 37.5 % (*χ*^2^ = 18.21, *p* = 0.001). The majority of the increase in concussions was attributed to sports-related injuries (Fig. 3). Concussions were documented most often in males (57.9 %). Two-fifths of all concussions occurred in patients 12–24 years of age (40.0 %). Sports-related injuries accounted for 29.9 % of all concussions, and football-related concussions accounted for nearly a fifth (19.4 %) of all sports-related concussions. Children less than 18 years of age accounted for 19.4 % of assault-related concussions.

## Discussion

Between 2007 and 2011, the number of diagnosed head traumas in U.S. EDs increased by 60 %; subgroup analyses noted that a substantial proportion of the increase was concentrated in children under the age of 11 and in adults over the age of 65. The increases in diagnosed head trauma may be explained partially by a combination of increased awareness by the public and increased diagnosis by healthcare practitioners [1, 10, 11, 23]. Providers may be more aware of the potential dangers of head trauma, thus increasing reporting. Similarly, parents and caretakers may be more aware of potential injuries to the children and elderly after sustaining a hit to the head from an object or a fall, thus leading to more patients presenting for evaluation. Alternatively, Marin and colleagues have postulated that increases in TBIs among young children and the elderly may be associated with these cohorts receiving fewer benefits from public health efforts, many of which focus on teenagers through middle-aged adults, specifically through legislation protecting student athletes and through motor vehicle laws [11].

The proportion of explicitly documented traffic-related injuries comprised nearly one-fifth (17 %) of all head traumas. This figure was in concordance with previously published studies, with another large nationally representative database study documenting motor vehicle traffic accidents as a mechanism of injury in approximately 16 % of TBIs in its sample in 2010 [11]. Moreover, traffic-related diagnosed head trauma increased by 17 % over the study period. Previous studies have documented a decline in traffic-related TBI hospitalizations in adolescents and other cohorts over time, and improvements to vehicle and traffic safety have been cited as a reasons for this decline [24, 25]. The decline in hospitalizations may signal a decrease in the severity of head traumas sustained in traffic-related crashes and demonstrate the positive impact of long-term public health efforts. However, this observation is tempered by the observed increase in traffic-related head traumas noted in this study. Although the cause for this increase is ultimately unknown, increased diagnosis by providers and awareness by the public may be contributing factors. Injury prevention efforts should continue to focus on at-risk groups, such as adolescent and young drivers.

Nearly a fifth of both assault-related head traumas (17 %) and assault-related concussions (19 %) occurred in children <18 years of age. These proportions suggest that a number of children may be presenting to U.S. EDs with assault-related head traumas, some of which are clinically mild. The mechanisms of assault-related injury are diverse and include blows to the head or other body parts, dropping, throwing, or shaking [26]. Most of the literature has generally focused on the characterization of moderate and severe TBI [27–30]. Mild TBIs, however, may also occur in assault injuries that do not directly involve the head, and it is likely that these injuries are underdiagnosed. Given the potential morbidity from untreated mild head trauma [4, 5, 31], healthcare providers should be vigilant for head traumas that may be secondary to other injuries in assault cases. An emphasis should be placed on ED and first responder education to detect cases of potential abuse and to evaluate for head trauma in such cases.

Nearly a third (30 %) of concussions was associated with a sport or recreational activity. Clear guidelines currently exist with respect to sports concussion management and opportunities for injury prevention exist through rule and culture changes in many sports [32]. Despite recent publicity and increased awareness, misconceptions regarding concussion symptoms and acceptable return to play guidelines persist among patients, coaches, and physicians [33–36]. On the field or court, rule enforcement and player protection are inconsistent, and unadvised play is still undertaken in light of athletic or peer pressure. A recently rejected settlement in a class action lawsuit against the National Collegiate Athletic Association (NCAA) highlights this issue and negotiations to overhaul NCAA concussion management policies are continuing [37, 38]. Sustained educational efforts and improved rule enforcement remain critical in protecting athlete health.

As the NEISS is designed specifically for injury surveillance purposes [13, 14], it provides information on injury mechanism, injury intent, and data on sports and recreational-related injuries not available in other administrative databases. However, the NEISS is difficult to compare to these databases due to the use of its own diagnostic coding system rather than ICD-9-CM codes [13, 18]. Rather than attempting to overhaul the NEISS, most efforts have focused on improving the accuracy and sensitivity of head trauma studies via epidemiological means. Studies by Xiang et al. and Thompson et al. sought to validate a proposed TBI case definition, utilizing the CDC ICD-9-CM TBI definition as a gold standard [15–17]. The sensitivity of TBI identification in these studies were high, however, a proportion of cases were not captured as TBIs in the NEISS [15, 16, 39, 40]. Thus, the term head trauma is used during the application of the case definition in this study, as this term is a more conservative and accurate descriptor of the cases captured by the proposed NEISS case definition.

This study has several limitations. Injuries treated in urgent care centers, physician offices, or on athletic fields that do not present to an emergency department are not captured by the NEISS database. The number of head traumas is likely to be underestimated, particularly minor head traumas, of which a substantial portion are treated in an outpatient setting [41]. Therefore, the results presented here may not be representative of all head traumas in the United States. The NEISS coding guidelines for coding internal organ injuries to the head is relatively nonspecific and may include cases where no explicit diagnosis of head trauma was made [18]. Furthermore, the NEISS database only includes the most severe diagnosis for each case, even if multiple diagnoses are present in ED records. Cases may have been excluded from this study when head trauma was, in fact, present [16]. The use of ICD-9-CM as a gold standard for validating the NEISS case definition used is also a limitation, as ICD-9-CM codes have long been criticized as a flawed means of disease classification, especially in cases of traumatic brain injury [40, 42, 43]. Development and assessment of a new bridging matrix between the NEISS and the ICD-10-CM coding system may be beneficial in developing a better working case definition for head injuries, as previous efforts with ICD-9-CM codes demonstrated promising results [17]. Despite these limitations, the strength of this study lies in its large, nationally representative sample and the concordance of its results with other studies utilizing large administrative datasets [11, 12].

## Conclusions

In summary, the increase in the number and rate of diagnosed head traumas treated in U.S. EDs warrants continued injury prevention efforts and public awareness. Currently, major injury prevention efforts have focused on improving motor vehicle safety and safe play in sports. The focus of these interventions targets a large proportion of the population, but also is less likely to include younger children and the elderly. As the public and medical community become more aware of the potential dangers of head traumas, regardless of severity, expanding injury prevention efforts to include these populations will become increasingly important, and may involve provider awareness concerning head trauma in child abuse, safe play for younger children, and product and home safety.

## Abbreviations

CDC: Centers for Disease Control and Prevention
CI: confidence interval
CPSC: U.S. Consumer Product Safety Commission
ED: emergency department
ICD-9-CM: International Classification of Diseases, Ninth Revision, Clinical Modification
NCAA: National Collegiate Athletic Association
NEISS: National Electronic Injury Surveillance System
NEISS-AIP: NEISS-All Injury Program
RR: relative risk
TBI: traumatic brain injury
